# A 3D-Printable Cell Array for In Vitro Breast Cancer Modeling

**DOI:** 10.3390/ijms252313068

**Published:** 2024-12-05

**Authors:** Ilaria Arciero, Silvia Buonvino, Valeria Palumbo, Manuel Scimeca, Sonia Melino

**Affiliations:** 1 Department of Chemical Sciences and Technologies, University of Rome “Tor Vergata”, Via Della Ricerca Scientifica 1, 00133 Rome, Italy; ilaria-29@libero.it; 2 Departmental Faculty of Medicine, UniCamillus—Saint Camillus International University of Health Sciences, Via di Sant’Alessandro 8, 00131 Rome, Italy; 3 Department of Experimental Medicine, University of Rome “Tor Vergata”, Via Montpellier 1, 00133 Rome, Italy; valeria.palumbo.25@students.uniroma2.eu (V.P.); manuel.scimeca@uniroma2.it (M.S.)

**Keywords:** fibroin, hydrogel, 3D cell models, osteoblast-like, cell migration

## Abstract

Breast cancer is the most common cancer and the second leading cause of cancer-related death in women. In advanced stages of the disease, breast cancer can spread and metastasize to the bone, contributing to malignant progression. The roles of tissue stiffness and remodeling of the tumor microenvironment are relevant in influencing cancer progression and invasiveness, but they are still poorly understood. In this study, we aimed to investigate the effect of bone tissue stiffness on breast cancer cell behavior, using 3D cell–biomaterial systems to model the in vivo conditions. For this purpose, we developed a 3D-printable cell array, which is a tunable and reproducible platform on small scale, where each compartment could mimic the physiological cancer environment with a shape and rigidity close to bone tissue. In this system, we observed that in the highly metastatic breast cancer line MDA-MB-231, embedded in PEG–silk fibroin (PSF) hydrogel spheres in the array’s cavities, increasing stiffness promotes trans-differentiation into osteoblast-like cells and the production of breast microcalcifications. Moreover, we also tested this 3D model as a platform to evaluate the cell response to the therapy, in particular, investigating the drug sensitivity of the cancer cells to chemotherapeutics, observing a decrease in drug resistance over time in the array.

## 1. Introduction

Currently, significant efforts are being directed toward developing models to better represent the vast complexity and heterogeneity of tumors. Cancer is inherently heterogeneous, driven by transformed cells that acquire abnormal characteristics, such as uncontrolled proliferation, resistance to drugs and cell death, enhanced angiogenesis, and the ability to evade the immune system, ultimately leading to metastasis [1]. Developing accurate tumor models is essential for understanding the unique characteristics of various cancers. As a result, three-dimensional (3D) cancer cell culture models have been widely employed, not only in the early stages of drug screening—where they have significantly improved the efficiency of antitumor drug development [2,3,4]—but also in studies related to gene expression, the development of immunotherapies, and the investigation of drug resistance mechanisms in cancer cells [5,6]. The development of efficient in vitro models, able to mimic the 3D micro-architecture of the tumor, can optimize both the times and costs of production of novel combined and precision cancer therapies and more accurately predict therapeutic efficacy [7,8,9,10,11].

The tumor microenvironment (TME) is a dynamic and ever-changing entity, playing a critical role in shaping cancer heterogeneity. It comprises diverse cellular and non-cellular elements, including immune cells (e.g., T cells, B cells, natural killer cells, macrophages, neutrophils, dendritic cells), fibroblasts, endothelial cells, cancer-associated fibroblasts (CAFs), adipocytes, the extracellular matrix (ECM), and soluble factors [12]. The complex interactions between tumor cells and these TME components are fundamental in driving the genetic and phenotypic plasticity observed in cancer [13]. Therefore, the replication of the tumor microenvironment using 3D in vitro models holds great potential for studying tumor formation and progression, which may, in turn, help in the discovery of new anti-cancer therapies. Spheroids and tumoroids embedded in cancer cells are particularly effective in recreating the spherical organization of in vivo tumor masses, accurately replicating the oxygen, nutrient, and drug gradients present within real tumors [4,14]. Furthermore, 3D tumoroids are invaluable for investigating dynamic processes such as cell migration and the invasiveness of certain solid tumors [14,15,16,17].

Among the various cancer types, breast cancer is the most prevalent malignancy among women worldwide (https://gco.iarc.fr/, accessed on 28 November 2024). It presents several alarming characteristics, such as a hypoxic microenvironment, patient-to-patient variability, intra-tumoral cellular heterogeneity [18], and a high propensity for metastasis, particularly to lymph nodes, bone, the liver, and the lungs [19]. Metastasis involves the dissemination of cancer cells, during which tumor cells acquire aggressive traits by undergoing an epithelial-to-mesenchymal transition (EMT). This process enables cells to invade distant organs from the primary tumor site [20]. During EMT, cancer cells gain mesenchymal traits, adopting a migratory phenotype and stem cell-like properties [21], which allow them to degrade the ECM via metalloproteinases and ultimately enter the bloodstream. Interactions with stromal cells further promote cancer cell invasion. Additionally, breast tumor tissues exhibit increased stiffness compared to normal mammary tissues, which enhances the migratory speed of tumor cells [22].

Bone is the most common site of breast cancer metastasis, with approximately 60 to 85% of patients with metastatic breast cancer developing bone metastases [23]. This is particularly prevalent in patients with triple-negative breast cancer, which is associated with a poor prognosis [24]. Bone matrix cells, including osteoblasts, secrete chemo-attractants such as CXCL12, osteopontin, receptor activator of nuclear factor kappa-B ligand (RANKL), and bone morphogenetic proteins (BMPs), which recruit cancer cells [25].

Hydrogel-based 3D culture systems provide a simplified yet effective approach to studying these complex processes and the molecular mechanisms underlying uncontrolled cell proliferation, migration, and metastasis [26]. Additionally, they can facilitate the prediction of patient responses to treatments and the development of personalized therapies [27]. Three-dimensional models have been developed to study breast cancer metastasis following extravasation to bone [28,29,30,31]. The roles of tissue stiffness and remodeling of the tumor microenvironment are relevant in influencing cancer progression and invasiveness [32], but they are still poorly understood. In this work, we have designed and optimized a handy 3D-printable array, using a bone matrix (BM) ink capable of mimicking bone stiffness, to create in vitro models of breast cancer, using a triple-negative MDA-MB-231 cancer cell line embedded in silk–fibroin hydrogel, to investigate the effects of the environment in terms of shape and rigidity on tumor growth, trans-differentiation, drug resistance, and tumor invasiveness.

## 2. Results

### 2.1. Fabrication of 3D-Printable MDABM Array

**Figure 1:**
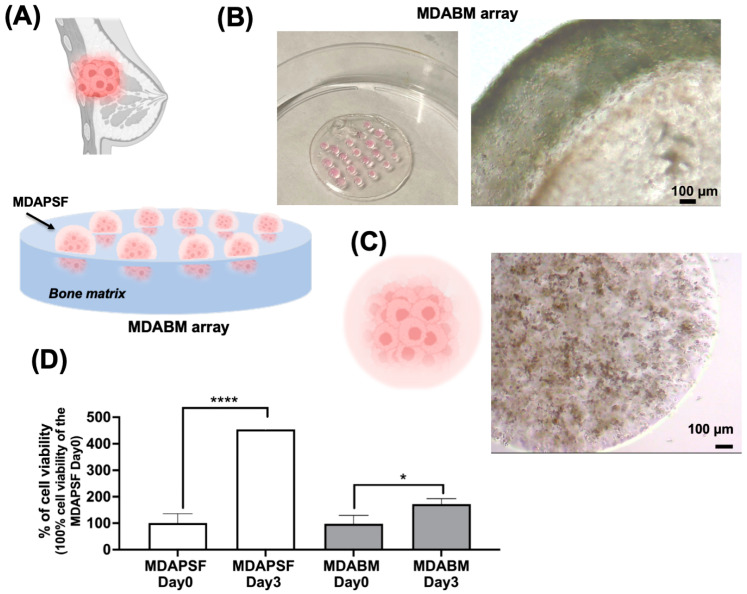
Three-dimensional breast cancer cell culturing in BM array. (**A**) A graphic representation of a breast tumor mass and a scheme of the array for 3D breast cancer in vitro modeling; (**B**) a digital image of the MDABM array prototype and a brightfield micrograph of MDAPSF (10 μL) in the BM array; (**C**) a graphic representation of MDAPSF and a brightfield micrograph of a 3 μL sphere in cell culture medium after 1 day of cell growth; (**D**) the cell viability WST-1 assay of MDAPSF and of MDABM on day 0 and after 3 days of cell growth. Each result was obtained from three or four independent biological replicates. The error bar indicates S.D. * *p* value ≤ 0.05, **** *p* value ≤ 0.0001. The scale bars are of 100 µm.

In this study, a 3D-printable breast cancer cell–BM array to model the physiological breast cancer environment was produced (Figure 1A,B). This array was produced using a commercial photopolymerizable bone matrix (BM) ink (by Stratasys) and highly metastatic breast cancer cells of the MDA-MB-231 cell line (MDA), which were embedded through direct photopolymerization in PSF hydrogel with 9% (*w*/*v*) of PEGDa into the BM array (MDABM) (Figure 1B), in order to simulate the stiffness of the physiological tissue.

MDA-MB-231 spheres in PSF hydrogel (MDAPSF) were also produced as a control without bone matrix through photopolymerization on the super-hydrophobic surface as described in our previous studies [14,17,33], using 9% (*w*/*v*) PEGDa (Figure 1C).

The cell proliferation of MDA-MB-231 cells in both the MDAPSF and MDABM arrays was assessed after 3 days of cell culture (Figure 1D), leading to an increase in the cell viability of MDAPSF over time of 353.2% ± 26.98 with respect to day 0, a much greater increase than what is observed in the MDABM array 74.41% ± 21.16.

The minor increase in cell proliferation in the array may be related to cell death, metabolic changes, and/or cellular trans-differentiation process.

### 2.2. Breast Cancer Cells’ Trans-Differentiation Toward Osteoblast-like Cells

**Figure 2:**
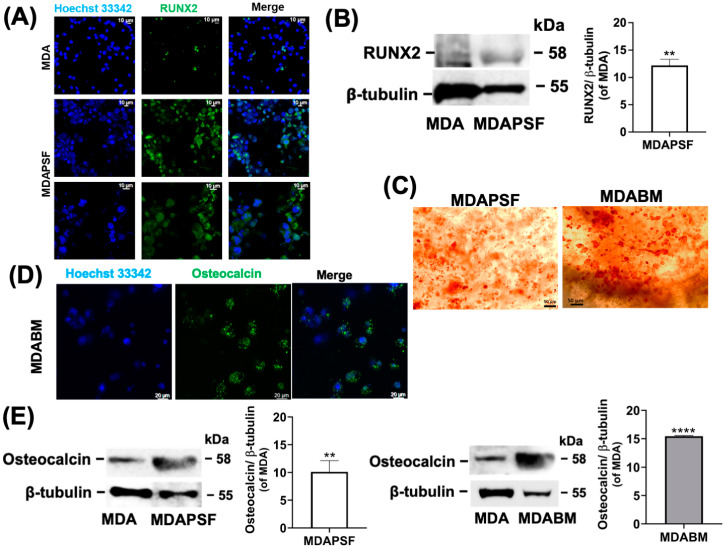
MDA cells’ trans-differentiation toward osteoblast-like cells. (**A**) Confocal fluorescence micrographs of MDAPSF (at cell density of 4 × 10^4^ cells/μL) after 3 days of cell growth and of MDA grown on tissue culture plate (TCP) (MDA); expression of RUNX2 was assessed through staining with Ab-RUNX2- Alexa-Fluor 488 nm (in green), and nuclei were stained in blue with Hoechst 33342. For MDAPSF, Z-stack were obtained by overlapping 183 slices, with zoom of 2.5, and by overlapping 52 slices, with zoom of 4.5; for MDA, zoom was 2. (**B**) Expression of RUNX2 according to Western blot analysis in breast cancer cells after 3 days of cell growth in 2D (MDA) and 3D growth systems (MDAPSF). For quantitative evaluation of protein expression of MDAPSF, each sample was obtained using 3 to 5 microspheres grown separately, so each sample was inherently mediated, and further, two independent experiments were performed. (**C**) Alizarin Red staining of MDA cells in PSF (MDAPSF) and PSFBM (MDABM) performed after 3 days of cell culture (at cell density of 10^4^ cells/μL). (**D**) Confocal fluorescence micrographs of MDABM (at cell density of 10^4^ cells/μL). The Z-stack was obtained by overlapping 103 slices (zoom 1.6). Osteocalcin was stained in green by Ab-Osteocalcin Alexa-Fluor 488 nm, and nuclei were stained blue; (**E**) osteocalcin expression assessed by Western blot analysis of MDA-MB-231 cells grown in 2D (MDA), MDAPSF, and MDABM after 3 days of cell growth. Protein expression in MDABM and MDAPSF was evaluated using 3 to 5 microspheres grown separately for each sample, and two and three independent experiments were performed, respectively. Error bars indicate S.D. ** *p* value ≤ 0.01, **** *p* value ≤ 0.0001. Scale bars are of 10, 20, and 50 μm.

The expression in MDAPSF of the protein RUNX2 (runt-related transcription factor 2), an earlier marker of osteogenic differentiation, was assessed by immunofluorescence staining and Western blot analysis (Figure 2A,B). Figure 2A shows confocal micrographs showing the presence of RUNX2, confirmed by the Western blot analysis (Figure 2B), which indicates about a 12-fold increase in RUNX2 expression in the MDAPSF system with respect to the 2D culture. The increased expression of RUNX2 in MDAPSF suggests that the stiffness of the hydrogel alone can induce the transformation of breast cancer cells into osteoblast-like cells, in agreement with our previous studies [9]. To assess the possibility of a trans-differentiation process of the breast cancer cells induced by increasing the stiffness of the cellular environment in the BM array, an Alizarin Red S (ARS) assay was performed, and the results are shown in Figure 2C.

**Figure 3:**
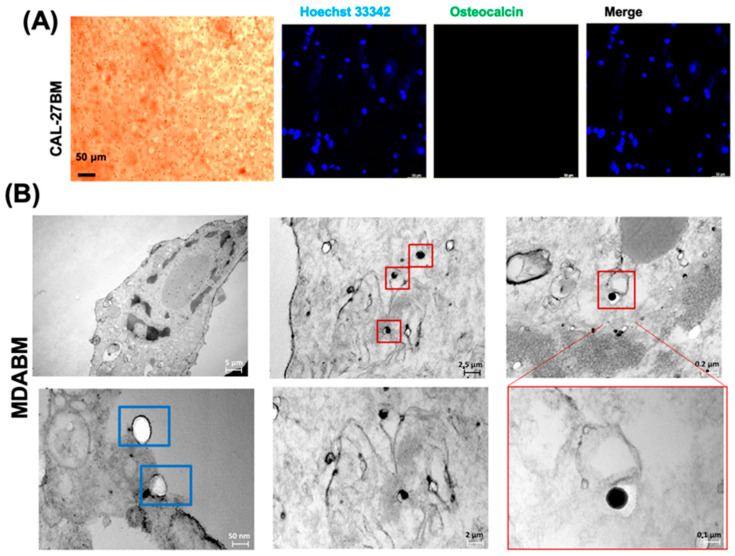
Effects of BM growth on CAL-27 cell line and TEM analysis of MDABM. (**A**) Alizarin Red staining of CAL-27BM (at cell density of 10^4^ cells/μL), performed after 3 days of cell culture, and confocal fluorescence micrographs of CAL-27BM after 3 days of cell growth in which osteocalcin is stained green and the nuclei are stained blue. (**B**) TEM micrographs of MDA MB-231 after 3 days of cell growth in BM array; in blue boxes and in red boxes are shown intracellular vesicles and calcified granules, respectively. Scale bars are of 50, 5, 2.5, 2, 0.2, and 0.1 μm and 50 nm.

Moreover, a significant increase in osteocalcin expression in a stiffness-dependent manner was also observable from the Western blot analysis, as shown in Figure 2E. In more detail, the increase in osteocalcin expression was about 10-fold in MDAPSF and 15-fold in MDABM compared to the 2D culture, after three days of cell growth. Figure 2D also shows confocal fluorescence micrographs of MDABM after three days of cell growth, stained with AlexaFluor 488 Ab-osteocalcin and Hoechst. The up-regulation of osteocalcin suggested the stiffness-dependent production of calcifications and physically induced trans-differentiation of the cancer cells into osteoblast-like cells, in agreement with our previously results [9]. The same treatment was performed on head–neck cancer cells of the CAL-27 cancer cell line. By contrast, no ARS-positive deposits or increase in osteocalcin expression (Figure 3A) was observed.

The trans-differentiation of the cells in the MDABM system into osteoblast-like cells was also confirmed by transmission electron microscopy (TEM) analysis. Ultrastructural investigations demonstrated the presence of vesicles containing electron-dense calcified granules in the cytoplasm of MDA-MB-231 cells after 3 days of cell growth in the BM array (Figure 3B). These granules appeared similar to the intracellular vesicles of osteoblasts [34]. Breast microcalcifications play a crucial role in early cancer diagnosis and are frequently associated with breast tumor development [35]. Ultrastructural energy-dispersive X-ray (EDX) microanalysis would be necessary to characterize the composition of microcalcifications; usually, calcium oxalate deposits are restricted to benign tissue, while calcium hydroxyapatite deposits occur both in benign and in malignant lesions [36].

### 2.3. Drug-Resistance of Tumor Cells in MDABM Array

**Figure 4:**
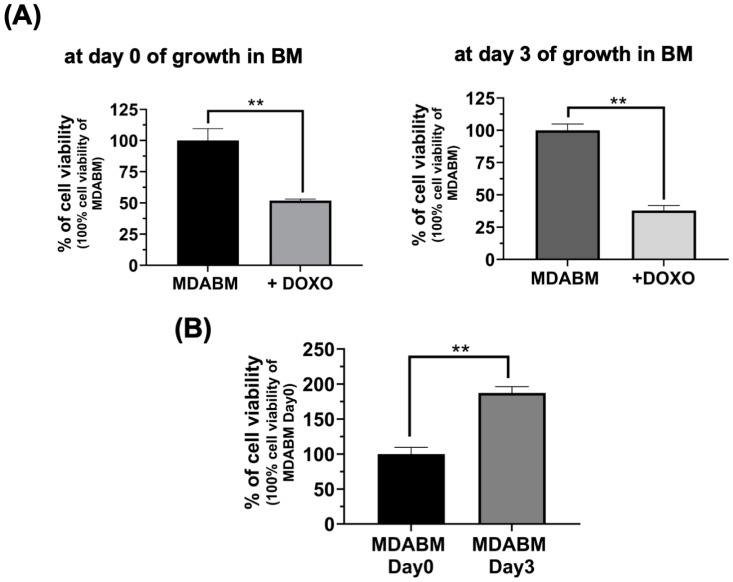
Drug resistance of MDABM. (**A**) Cell viability assay of MDABM treated with 2 μM of doxorubicin for 24 h (+DOXO) on day 0 and after 3 days of cell growth. (**B**) Cell viability assay of MDABM on day 0 and after 3 days of cell growth. Each result was obtained using three independent biological replicates. Error bars indicate S.D. ** *p* value ≤ 0.01.

The biochemical and physical features of the tumor microenvironment are relevant not only for the induction of cells trans-differentiation, but also for their drug-resistance [6,14,37]. Therefore, we investigated the doxorubicin-resistance of the cancer cells grown in the BM array. The cell viability performed after 24 h of treatment with 2 µM of doxorubicin (DOXO) (Figure 4A) shows that the cancer cells grown for three days in the BM array had lower cell viability (37.88% ± 3.8) compared to the cancer cells treated with doxorubicin immediately after MDABM production (51.87% ± 1.16). This major sensitivity of the cells grown three days in the MDABM system may be in agreement with the observed trans-differentiation process of cancer cells in osteoblast-like cells, also considering the increase in cell viability, due to cell proliferation, in the absence of doxorubicin after three days (187.3% ± 9.1) (Figure 4B). The cell viability of MDA-MB-231 on a tissue culture plate (TCP) in the presence and in the absence of doxorubicin is shown in Figure S1.

### 2.4. Effects of Osteoblast-like Tumor Cells on Migration of Breast Cancer Cells in 2D Cultures

**Figure 5:**
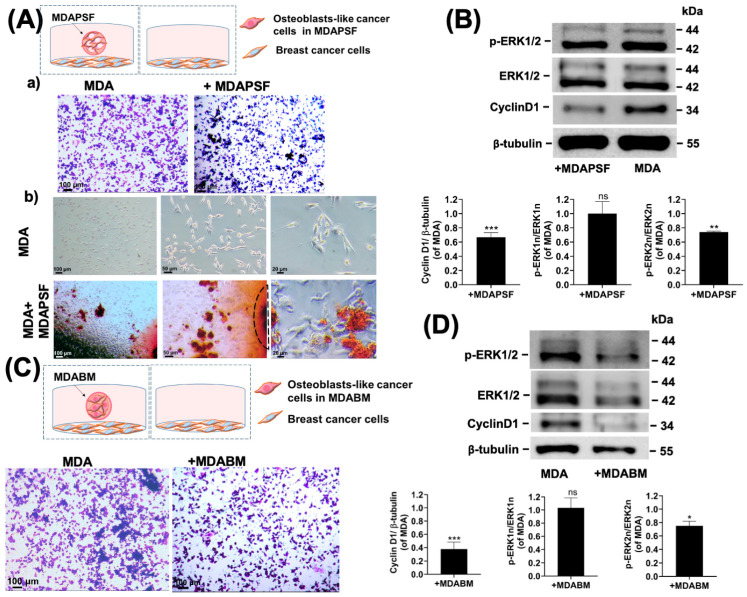
Effects of biochemical factors released from trans-differentiated osteoblast-like tumor cells on MDA-MB-231 cells. (**A**) Scheme of the experimental setup: (**a**) trans-well migration assay and (**b**) Alizarin Red staining of 2D MDA cells cultured for 48 h in absence (MDA) and presence of MDAPSF (+MDAPSF). (**B**) Western blot analysis of cyclin D1 and p-ERK1/2 expression MDA and +MDAPSF; cyclin D1 and p-ERK1/2 expression was mediated by three independent biological replicates. (**C**) Scheme of experimental setup and trans-well cell migration assay of 2D MDA cells cultured for 48 h in absence and presence of MDABM (+MDABM). (**D**) Expression of cyclin D1 and p-ERK1/2 assessed through Western blot analysis in MDA and +MDABM. Expression of cyclin D1 and p-ERK1/2 was mediated by three independent biological replicates. Error bars indicate S.D. ns = non-significant, * *p* value ≤ 0.05, ** *p* value ≤ 0.01, *** *p* value ≤ 0.0005. Scale bars are of 100, 50, and 20 μm.

It is known that the trans-differentiation of breast cancer cells into osteoblast-like cells can have effects on tumor invasiveness and the malignant progression of nearby breast cancer cells [38]; however, it is not clear if osteoblast-like cancer cells can induce changes in the invasiveness of nearby cancer cells through the release of biochemical factors (protein, exosomes, etc.) and/or change the physical parameters of the TME, such as the stiffness, through the release of calcium deposits. To assess this hypothesis, we used the MDAPSF and MDABM systems as tools to enable the rapid and easy trans-differentiation of breast cancer cells and to evaluate the different effects, as schematized in Figure 5A,C.

The effects of the biochemical factors released by trans-differentiated cells on the cancer cells were investigated by co-culturing MDAPSF spheres on day four of cell growth with MDA-MB-231 cells seeded on TCP (Figure 5A). After 48 h of cellular co-culture, a small reduction in cancer cell invasiveness was observed with respect to the cancer cells cultured in the absence of MDAPSF spheres (Figure 5A(a)). Moreover, the Alizarin Red assay showed the presence of calcium deposits both in the MDAPSF spheres and on the cancer cells very near them, as shown in Figure 5A(b). Therefore, the reduced invasiveness may be related to trans-differentiation induction by the MDAPSFs due to diffusible biochemical factors. The reduced invasiveness was also confirmed by the down-regulation of p-ERK2 and cyclin D1 expression in the cancer cells co-cultured with MDAPSF, as shown in the Western blot analysis in Figure 5B. The same experiments were performed for co-culturing the breast cancer cells in the presence of MDABM spheres, previously cultured for three days in the BM array (as schematized in Figure 5C). In agreement with the effects of the MDAPSF spheres, the invasiveness of the breast cancer cells was reduced by co-culturing (Figure 5C). Moreover, a major decrease in cyclin D1 and p-ERK2 expression compared to the MDAPSF co-culture was observed (Figure 5D). A decrease in both cyclin D1 and p-ERK1/2 expression has been associated with a decrease in cell proliferation and migration [39,40]. Therefore, these results indicate that diffusible biochemical factors released by osteoblast-like cells can stimulate the trans-differentiation of other cancer cells without increasing their invasiveness, which could be influenced by physical factors, such as an increase in local stiffness in the TME.

### 2.5. Effects of Physical Interaction with Osteoblast-like Tumor Cells on Migration of Breast Cancer Cells in 3D Cultures

**Figure 6:**
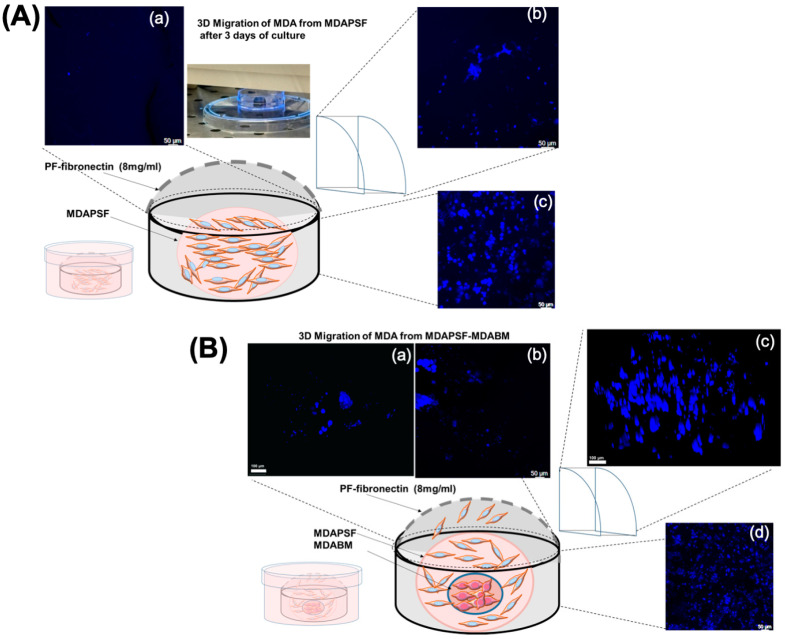
Three-dimensional cell migration assay of MDA cells. A scheme and confocal micrographs of the 3D cell migration assay of MDAPSF (at a cell density of 10^4^ cells/μL) in the absence (**A**) and in the presence (**B**) of MDABM (MDAPSF-MDABM). MDAPSF or MDAPSF-MDABM was embedded in the outer gel made of PF–fibronectin 8 mg/mL and cultured for 3 days. The nuclei were stained blue with Hoechst 33342 (nuclei staining of live cells). The Z-stacks of (**A**) were acquired by overlapping the 79 (**a**), 169 (**b**), and 199 (**c**) slices, and in (**B**) by overlapping the 79 (**a**), 79 (**b**), 175 (**c**), and 191 (**d**) slices. The scale bars are of 50 and 100 μm.

Given the critical relevance of the three-dimensional microenvironment in cell invasion, we analyzed the effects of the physical factor that occurs due to the increased stiffness of the rigid core of trans-differentiated cells (MDABM) on cancer cell invasiveness by performing a new 3D cell migration assay. We evaluated breast cancer cell migration in a 3D hydrogel system in the presence and in the absence of MDABM, as schematized in Figure 6. MDA-MB-231 cells were embedded in PSF hydrogel at a density of 10^4^ cells/μL either in the absence or in the presence of MDABM on day 3 of cell culture. After the photopolymerization of MDAPSF with and without MDABM, an outer hydrogel of PEG–fibrinogen (PF)–fibronectin with lower stiffness hydrogel of about 50 Pa [15,41] was produced. The 3D systems were cultured in cell culture medium for three days, and after staining of the live cells with Hoechst 33342, the cancer cell invasion of the outer gel was evaluated via confocal microscopy. By comparing confocal micrographs of the outer gels in the presence and in the absence of the MDABM (Figure 6), a higher presence of MDA cells in the outer gel was observed in the presence of MDABM (Figure 6B). This result suggests that physical interactions with osteoblast-like cells enhance the migration of MDA cells in 3D culture systems and highlights the relevance of physical factors, such as stiffness and direct interaction, in the increase in cancer cells’ ability to migrate/invade.

## 3. Discussion

Breast cancer is the most common cancer and the second leading cause of cancer-related death in women [42]. In advanced stages of the disease, breast cancer can spread and metastasize to the bone, contributing to malignant progression [38,43]. We produced a 3D BM array, with tunable physical properties due to the presence of PSF hydrogel, as a versatile system embedding breast cancer cells of the MDA-MB-231 cell line for the in vitro modeling of breast cancer [23,25].

Recent studies have shown that tumor cells can be reprogrammed into cancer stem cells (CSCs) capable of differentiation when cultured on a hydrogel matrix [44]. Furthermore, other studies have highlighted that physical stimuli within the TME, such as stiffness, can drive breast cancer cells to undergo EMT, followed by osteogenic differentiation [14,45]. During this process, breast tumor cells acquire osteoblastic properties, including the expression of osteoblastic transcription factors (such as RUNX2) and osteoblastic proteins (such as osteocalcin), which are involved in microcalcification and bone matrix formation [35]. Previous studies on biopsies have shown the formation of breast osteoblast-like cells (BOLCs), originating from breast epithelium–mesenchymal transformation, that are able to produce microcalcifications made of hydroxyapatite (HA) [38]. However, the impact of this physically induced trans-differentiation (PiT) on disease progression remains unclear.

The BM array simulates bone tissue stiffness, allowing the investigation of the effects of physical stimuli on tumor proliferation, differentiation, drug resistance, migration, and invasiveness. In MDAPSF, the expression of RUNX2 and osteocalcin was detected, demonstrating that stiffness of the PSF hydrogel of about 13 kPa alone can induce the transformation of breast cancer cells into osteoblast-like cells after just three days of growth. Altogether, osteocalcin expression, the presence of calcium deposits, and the osteoblast-like morphology revealed that the increased stiffness provided by the BM array promoted the production of microcalcifications and improved the trans-differentiation of MDA cells into osteoblast-like tumor cells, to a greater extent than the 3D PSF spheroid.

Moreover, several studies have demonstrated that osteoblast-like features enhance the propensity of breast cancer cells to proliferate, metastasize to bone, and increase their resistance to the multidrug therapies commonly used in breast cancer treatment [35,38,45,46]. Therefore, we investigated the doxorubicin resistance of trans-differentiated cancer cells in MDABM system, observing a decrease in drug-resistance in a time-dependent manner from the cell growth in the BM array. This finding aligns with our recent publication [9], where we highlighted an inverse correlation between Multidrug Resistance (MDR) glycoprotein P (Pgp) expression and the stiffness of tumoroids, which was in keeping with the major sensitivity to doxorubicin in MDAPSF models with higher stiffness compared to those with lower stiffness. These results underscore the importance of integrating a biomimetic physical microenvironment into any in vitro model and demonstrate that the stiffness of the tumor microenvironment significantly influences the response of breast cancer cells to chemotherapy. Although the cells in MDABM exhibit reduced drug resistance, the production of osteocalcin and other biochemical factors (e.g., Ca^2+^, MMPs, miRNAs) could stimulate un-trans-differentiated cancer cells to increase their proliferation and the production of MMPs, with a reduction in matrix stiffness and, consequently, an increase in cell migration and metastasis.

We assessed whether trans-differentiation into osteoblast-like cells could influence the tumor invasiveness of un-trans-differentiated breast cancer cells, evaluating the effects of the biochemical factors described above on breast cancer cells in 2D culture, and the effects of physical factors given by the increased stiffness of the rigid core of trans-differentiated cells on cancer cell invasion in 3D culture, by performing a 3D cell migration assay.

Our results showed that breast osteoblast-like cells were able to induce, through the release of biochemical factors into the culture medium, the osteoblastic trans-differentiation of co-cultured breast cancer cells in 2D cell culture systems, leading to a decrease in their proliferation and migration, as evidenced by the down-regulation of p-ERK2 and cyclin D1 expression. In contrast, in 3D culture systems, the physical interaction with osteoblast-like cells increased the migration of MDA-MB-231 cells. These findings underline the significant role of physical factors, such as stiffness, in promoting cancer cell invasion in 3D tumor models, suggesting the need for studies on cancer cells invasiveness, which are mainly focused on the effects of biochemical factors, to adopt a wider scope. The ability of osteoblast-like cells to promote the invasiveness of breast cancer cells in 3D culture systems aligns with ex vivo studies, where the presence of BOLCs in human biopsies was associated with a high propensity for cancer cells to develop bone metastases within five years after diagnosis [47]. The BM array is able to integrate two 3D-printable materials with different stiffness, allowing precise control over the mechano-physical cues that cells experience when embedded in the 3D system. This system can enable us to analyze the effects of various bone extracellular microenvironments on different cell types, effectively mimicking cell behavior in native tissues and under different pathological conditions. Moreover, the 3D cell array can represent an in vitro system useful to better mimic the physiological tumor microenvironment, enabling the study of the effects of cancer cell trans-differentiation on surroundings cells and the development of advanced 3D co-culture models of breast cancer. Furthermore, our study represents a starting point to validate the use of the BM printable ink for the production of 3D personalized arrays, facilitating the assessment of anti-tumor drug responses in the development of breast cancer therapies.

## 4. Materials and Methods

### 4.1. Cell Cultures and Hydrogel-Based Tumoroid Production

For the experiments, highly metastatic triple-negative breast cancer cells of the MDA-MB-231 cell line and the human oral squamous cell carcinoma of the head and neck (OSCC) cell line CAL-27 (Gibco, Milano, Italy) were used. Cells were cultured using Dulbecco’s Modified Eagle Medium (DMEM) with high glucose (Gibco, Thermo Fisher Scientific, Milan, Italy), supplemented with 10% of Fetal Bovine Serum (FBS) (*v*/*v*) (Gibco, Thermo Fisher Scientific, Milan, Italy), 1% of penicillin–streptomycin (*w*/*v*) (Sigma-Aldrich, Milan, Italy), and 2 mM L-Glutamine solution (Gibco, Thermo Fisher Scientific, Milan, Italy). MDA-MB-231 and CAL-27 cells were resuspended in PEG–silk fibroin (PSF) hydrogel precursor solution, obtained as previously described [14,48], to produce PSF microspheres. The cells were resuspended at a cell density of 10^4^ cells/μL in PSF precursor solution composed of 70 µL of PEG–silk fibroin, 6 mg/mL fibroin, 30 μL of 30% *w*/*v* PEGDa 10 kDa, and 1% *v*/*v* of a photo-initiator stock solution containing 10% *w*/*v* Irgacure^®^ 2959 (Ciba Specialty Chemicals, Basel, Switzerland) in 70 vol% ethanol, for a total volume of 100 μL. Drops of 3–10 μL of the precursor solution containing cells were put on a nanostructured super-hydrophobic surface of polydimethylsiloxane (PDMS) obtained based on the protocol described by Patent LDO0252 [49]. The drops were exposed to UV light (365 nm, 4–5 mW cm^–2^) for 2 min. PSF has been extensively characterized in our previous works [14,48], and a rheometric analysis of the hydrogel was performed, determining a shear storage modulus (G′) of 13.4 kPa.

### 4.2. BM Array Production and 3D Cell–BM Array Culturing

The 3D-printable BM array was created using bone matrix (BM) ink (Stratasys, Minneapolis, MN, USA) by photopolymerizing the ink in a circular mold under UV light (365 nm, 4–5 mW cm^−2^) for one minute. Array cavities were formed by placing 10 µL water droplets on the hydrophobic ink before polymerization. MDA-MB-231 and CAL-27 cells (at a density of 10⁴ cells/µL) were embedded in PSF spheres and photopolymerized directly within the BM array cavities to create 3D cell–BM cultures. The array’s CAD design, compatible with the Stratasys J750 Digital Anatomy 3D Printer, was created using Blender software (Blender version 3.3, Amsterdam, The Netherlands).

### 4.3. Cell Viability Assay

The cell viability of MDAPSF and of MDABM was assessed on day 0 and after 3 days of cell growth. The cell viability of MDABM was also assessed after 24 h of treatment with 2 μM of doxorubicin (DOXO), added into the cell culture medium on day 0 and on day 3 of growth in the BM array. To assess the cell viability, a WST-1(4-[3-(4-lodophenyl)-2-(4- nitrophenyl)-2H-5-tetrazolium]-1,3-benzene disulfonate (Cell Proliferation Reagent WST-1, Roche, Mannheim, Germany) assay was used [50]. Briefly, the cell culture medium was replaced with fresh DMEM with high glucose without phenol-red (Gibco, Italy) containing tetrazolium salt WST-1 (5% *v*/*v*). The samples were incubated for 4 h at 37 °C and 5% CO_2_, and then, the absorbance of the medium was evaluated using a microplate reader at a wavelength of 450 nm.

### 4.4. Alizarin Red S Staining

Alizarin Red S staining to detect calcium-rich deposits was performed on MDAPSF, MDABM, and CAL-27BM after 3 days of cell growth. Moreover, Alizarin Red staining was performed on MDA-MB 231 cells seeded on TCP at a cell density of 10^4^ cells/cm^2^ in a 12-well plate and, 3 h after seeding, co-cultured for 48 h in the presence of MDAPSF (three spheres of 8 μL, cell density of 10^4^ cells/µL) previously grown for 4 days. The samples were fixed with 4% paraformaldehyde (PFA), stained with Alizarin Red S solution pH 4.1 (Sigma-Aldrich, Italy) for 10 min, and washed twice with H_2_O_dd_, according to the specific protocol. The Alizarin Red staining was assessed via optical microscopy using a Primovert Zeiss microscope (Zeiss, Jena, Germany) and a BA310 Motic microscope (Mitoc, Hackensack, NJ, USA).

### 4.5. Confocal Fluorescence Microscopy

MDAPSF, MDABM, and CAL-27BM were analyzed via immunofluorescence staining. After 3 days of cell culture, the cells were fixed with PFA 4% in PBS for 30 min at room temperature, then permeabilized with 0.3% Triton X-100 for 5 min and maintained in blocking buffer (10% *v*/*v* FBS, 0.1% *v*/*v* Triton X-100, and 1% *w*/*v* glycine in PBS) overnight at 4 °C. Afterward, the samples were incubated overnight at 4 °C with 1:200 *v*/*v* Ab-osteocalcin primary antibody (Thermo Fisher Scientific, Invitrogen, Waltham, MA, USA) in PBS with 1% albumin with 20 mM Gly solution, followed by 4 h of incubation with an Alexa fluorochrome 488 nm-conjugated secondary antibody in 20 mM Gly-PBS at room temperature (Thermo Fisher Scientific, Invitrogen, USA). MDAPSF and MDA cells seeded on TCP were also stained using Ab-RUNX2 Alexa-Fluor 488 nm. Cell nuclei were stained with Hoechst 33342 (Sigma-Aldrich, Italy). Confocal fluorescence analysis was performed using a Stellaris Leica microscope platform (Leica, Wetzlar, Germany).

### 4.6. Transmission Electron Microscopy (TEM)

The MDAPSF spheres inside the BM array were removed from the array and processed for the TEM analysis. The MDAPSF spheres were fixed in 4% PFA and post-fixed in 2% osmium tetroxide following a previously published protocol [51]. After washing with 0.1 M phosphate buffer, the samples were dehydrated by a series of incubations in 30%, 50%, and 70%, ethanol. Dehydration was continued through incubation steps in 95% ethanol, absolute ethanol, and hydroxypropyl methacrylate; then, the samples were embedded in Epon resin (Agar Scientific, Stansted Essex, UK) [51]. Sections were observed with a Morgagni 268D transmission electron microscope (FEI, Hillsboro, OR, USA).

### 4.7. Assessing Protein Expression via Western Blot Analysis

Proteins were extracted from MDA-MB-231 cells grown in 2D and 3D culture systems (MDAPSF and MDABM) using 50 μL of sample buffer for SDS-PAGE for each sample. The samples were vortexed and boiled for 5 min, centrifuged for 5 min at 10,000 rpm, and stored at −20 °C. For SDS-PAGE, 12% polyacrylamide gels were used and the electro-blotting was performed on PVDF membranes (Amersham Hybond P, Merk, Milan, Italy). After blocking the membranes, they were probed overnight at 4 °C with primary monoclonal antibodies: Ab-osteocalcin mouse (MA1-20786, Thermo Fisher Scientific, Invitrogen, Rodano, Italy); Ab-cyclin D1 rabbit (2922S, Cell Signaling Technology, Italy); Ab-p-ERK1/2 rabbit (Anti-p-ERK1 (pThr202/pTyr204) and ERK2 (pThr185/pTyr187) (ab136926 Abcam, Milano, Italy); Ab-ERK1/2 rabbit (M5670, Sigma-Aldrich, Italy); and Ab-RUNX2 mouse (ab76956, Abcam, Cambridge, UK). ERK1/2 detection, after p-ERK1/2 probing, was performed by re-probing the membrane after stripping it with 2% SDS and 100 mM β-mercaptoethanol at 40 °C for 60 min and washing it with PBS. Then, the membranes were incubated with the secondary antibodies rabbit (dilution 1:3000) (7074S, Cell Signaling Technology, Milano, Italy) and mouse (7076S, Cell Signaling Technology, Italy) for 4 h at room temperature. Ab-β-tubulin-HRP (AB21058, Abcam, Cambridge, Italy) was also used to check the protein loading. A Super Signal West Pico kit (Thermo Scientific, Italy) was used to visualize the signal, followed by exposure to a Fluorchem Imaging system (Alpha Innotech Corporation—Analitica De Mori, Milan, Italy). Western blot (WB) analysis was performed on MDA-MB-231 cells seeded at a cell density of 10^4^ cells/cm^2^ on a 24-well plate and, 3 h after seeding, co-cultured for 48 h in the presence of MDABM (sphere of 10 μL at a cell density of 10^4^ cells/μL) previously grown for 3 days. WB analysis was also performed on MDA-MB-231 seeded at a cell density of 10^4^ cells/cm^2^ on a 12-well plate and, 3 h after seeding, co-cultured for 48 h in the presence of 3 MDAPSF spheres (spheres of 8 μL at a cell density of 10^4^ cells/μL) previously grown for 4 days. WB analysis of 3D MDAPSF and of MDABM was performed after 3 days of culture, and the quantitative evaluation of protein expression was obtained using 3–5 microspheres for each sample, therefore also being inherently mediated.

### 4.8. Trans-Well Migration Assay

A trans-well migration assay was performed on MDA-MB-231 cells seeded at a cell density of 10^4^ cells/cm^2^ on a 24-well plate and, 3 h after seeding, co-cultured for 48 h in the presence of MDABM (sphere of 10 μL at a cell density of 10^4^ cells/μL) previously grown for 3 days. The assay was also performed on MDA-MB-231 seeded at a cell density of 10^4^ cells/cm^2^ on a 12-well plate and, 3h after seeding, co-cultured for 48 h in the presence of 3 MDAPSF spheres (spheres of 8 μL at a cell density of 10^4^ cells/μL) previously grown for 4 days. After 48 h, a trans-well migration assay of MDA-MB-231 cells grown in the absence (MDA) and in the presence of MDABM (MDA + MDABM) or of MDAPSF (MDA + MDAPSF) was performed. Cell migration was assessed using 8 μm-pore-size Falcon TM Cell Culture Inserts (Thermo Fisher Scientific, Milan, Italy). Cells were detached and seeded in the upper chamber of the inserts in serum-free DMEM (10^5^ cells/insert). Complete DMEM (with 10% *v*/*v* of FBS) was added to the lower chamber to attract the cells. After incubation for 6 h at 37 °C and 5% CO_2_, a water-wetted cotton swab was used to remove cells from the upper surface of the membrane, and migrated cells on the other side of the membrane were fixed and stained with a solution of 6% (*v*/*v*) glutaraldehyde and 0.5% (*w*/*v*) crystal violet in H_2_O_dd_ for 15 min. The inserts were then washed 3 times with water, and finally, the air-dried membranes were analyzed using a Primovert Zeiss optical microscope.

### 4.9. Three-Dimensional Cell Migration Assay

Herein, we have developed a protocol to perform a 3D cell migration assay as follows. MDABM (10 μL) was produced with a cell density of 10^4^ cells/μL and cultivated for 3 days. MDABM was then embedded in another MDAPSF sphere (40 μL) containing MDA-MB-231 cells at a cell density of 10^4^ cells/ μL. As controls, samples with only MDAPSF, without embedding MDABM, were produced. After 24 h of growth, the 3D constructs (MDAPSF-MDABM or MDAPSF) were incorporated into an external and less stiff hydrogel (of about 50 Pa) made of PEG–fibrinogen (PF)–fibronectin. The outer gel was produced following the protocol developed by Ivanir et al. as previously described [10]. Briefly, a precursor solution containing PEG–fibrinogen (PF)–fibronectin (final concentration: 8 mg/mL of fibrinogen) and 1% *v*/*v* of a photo-initiator stock solution containing 10% *w*/*v* Irgacure^®^ 2959 (Ciba Specialty Chemicals, Basel, Switzerland) in 70 vol% ethanol was produced. A total of 150 μL of this solution was photopolymerized to encapsulate the MDAPSF-MDABM (or MDAPSF) using a circular mold, and we exposed the samples to UV light (365 nm, 4–5 mW cm^–2^) for 5 min. After 3 days of culture, live cells were stained with Hoechst 33342 (Sigma–Aldrich, Italy) (nucleus staining of live cells) and the samples were analyzed by confocal microscopy using a Stellaris Leica microscope platform.

### 4.10. Statistical Analysis

Data were obtained from three or more independent biological replicates and were analyzed for each variable using a parametric *t*-test. GraphPad Prism version 8.0 for Windows (GraphPad software, San Diego, CA, USA) was used to quantify the data and perform the statistical analysis. A *p*-value < 0.05 was considered statistically significant. The data are presented as mean ± standard deviation (S.D.).

## Data Availability

The data will be made available on request.

## Supplementary Materials

The following supporting information can be downloaded at: https://www.mdpi.com/article/10.3390/ijms252313068/s1.
