# Supplementary material for: A 3D-Printable Cell Array for In Vitro Breast Cancer Modeling
Source: Int J Mol Sci. 2024 Dec 5;25(23):13068. doi: 10.3390/ijms252313068 (PMC11642152; doi:10.3390/ijms252313068)
Supplement: Supplementary file 1 [file ijms-25-13068-s001.zip › Supplementary Materials Arciero et al 2024 IJMS.pdf]

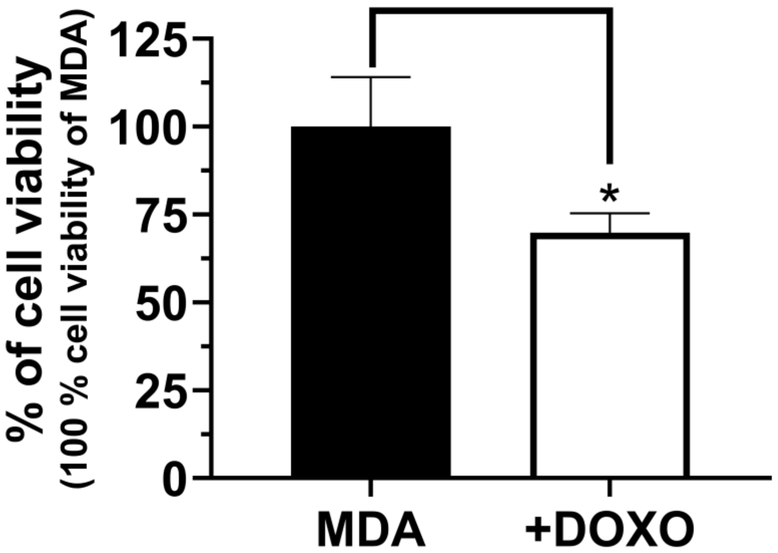

Figure S1. Effect of doxorubicin on the cell viability of MDA-MB-231 cancer cell line. Cell viability assay of MDA grown on TCP treated with 2 μM of doxorubicin for 24 h (+DOXO) at day 0 and after 3 days of cell growth. Error bars indicate S.D. * p value ≤ 0.05.
